# A Rare Case

**Published:** 1896-01

**Authors:** H. L. Ambler

**Affiliations:** Cleveland, O.


### A Rare Case.

BY H. L. AMBLER, M.D., D.D.S., CLEVELAND, O.

Abstract of paper read at the American Dental Association, August, 1895.

A young man, aged seventeen years, has both superior lateral incisors missing and no outward signs of their being imbedded in the jaw.

The inferior denture has the proper number of teeth, but both of the central incisors are remarkably small, perhaps one-fourth the size they should be; still, they are of good form and structure, firmly fixed in the alveolus, and the gingiva indicates well-formed roots, about in proportion to the crowns. The young man's father, mother and grandmother, who are very intelligent people, and have a dentist in the family, state that they are positive that the temporary central incisors were shed, and that these two small teeth erupted in their places.

Both superior and inferior temporary dentures were normal. His mother needs a left superior lateral incisor to complete a full denture; the father's is complete. The grandfather and grandmother, on both the father's and mother's side, had the full number of teeth. The only abnormality we could trace, except the mother's, was that the grandmother retained the left superior temporary cuspid until she was fifty-three years of age, at which time a permanent cuspid, of fair form and size, erupted and is still in place after a lapse of twenty-three years.

